# Supplementary material for: Identifiability and numerical algebraic geometry
Source: PLoS One. 2019 Dec 13;14(12):e0226299. doi: 10.1371/journal.pone.0226299 (PMC6910699; doi:10.1371/journal.pone.0226299)
Supplement: Attachment — Submitted filename: bates-review.pdf [file pone.0226299.s001.pdf]

The article “Identifiability and numerical algebraic geometry” is an engrossing and insightful article applying state-of-the-art methods in numerical algebraic geometry to significantly enlarge our toolbox for parameter identifiability analysis. There are a few passages that should be clarified and some typos, but after minor revision, I strongly recommend publication.

Edits

- Abstract: “such as a mathematical” delete “a”.
- Abstract: Polynomial or rational ODE’s should be mentioned so that complex numbers make sense.
- P. 3: Monic is generally defined for univariate polynomials, so its use here should be clarified. Same for “non-monic”.
- P. 4 (Method 1): The use of p as specializations of q is a bit unclear. At first it seems like q are themselves complex numbers.
- P. 4: Explain how to interpret k_{ij} as flow from j to i.
- P. 4 (Figure 1): There are two in-edges to state 1 that do not have obvious interpretation.
- P. 5 (Proposition 2.5): Is it necessary to specify the closure, rather than just the projection pre-closure? If so, you should specify Zariski (Euclidean, etc.) closure.
- P. 7 (Method 2): Example 2.6 would have terminated at $r = 2$ based on the last line of this algorithm. Perhaps you need that the coranks are also constant from step to step.
- P. 8 (Definition 2.9): The use of “multi-valued functions” doesn’t quite work. In partuclar, the equation $\xi \circ c = g$ has a multi-valued fn on the LHS, and a single-valued on the RHS.
- P. 8 (Problem 2.11): The phrase “given by the functions...” is a bit unclear. Are these the elements of the tr.basis?
- P. 8 (Prop 2.12): Given this setup, wouldn’t f be *globally* identifiable? If not, can you give an illustrative example?

- P. 9: The sentence beginning "While this information...." is difficult to parse.

- P. 11: For readers who are not familiar with the trace test, it will be difficult to understand from this description. In particular, the function $p_z(t)$ is not clearly defined, and it's not clear that summation is coordinatewise. I would recommend including a clarifying picture (see, for example, Figure 1 in Leykin, Rodriguez, Sottile). Alternatively, skip an explanation of the trace test and just include the reference.

- P. 13: Replace "yielding" with "yielding the result that" or "indicating that".

- P. 13: "is the number of which correspond with the same input-output" -¿ " is the number of points corresponding to the same input-output."

- P. 14 (Section 4, Paragraph 1): "every function of the parameter" -¿ "parameters"

- P. 15: In the sentece beginning "Hence", the conclusion is not immeditely obivous. Is it because all parameter values are different than in p?

- P. 17 (Method 5): "Use homotopy sampling to additional" -¿ "to find additional"

- P. 21: "in these 16 unidentifiable parameters" (maybe add "in these latter 16" to differentiate from the first set.
