# Supplementary material for: Identifiability and numerical algebraic geometry
Source: PLoS One. 2019 Dec 13;14(12):e0226299. doi: 10.1371/journal.pone.0226299 (PMC6910699; doi:10.1371/journal.pone.0226299)
Supplement: Attachment — Submitted filename: Response to Reviewers.pdf [file pone.0226299.s002.pdf]

29 September 2019

Professor Fang-Bao Tian
Academic Editor
PLoS ONE

Dear Professor Tian,

Thank you for the referee report of 20 September 2019. We thank the referees for the helpful comments on our manuscript *Identifiability and numerical algebraic geometry* [Manuscript ID PONE-D-19-15150]. We appreciate their suggestions, which we have incorporated into a revised version of the manuscript. We have also addressed the two journal requirements described in the referee report.

We hope that you will agree that the revised manuscript is a significant improvement over the original version. Below this message are our point-by-point responses.

Yours sincerely,

Dan Bates, Jonathan Hauenstein, and Nikki Meshkat

——————— Journal Requirements ———————

1. Please ensure that your manuscript meets PLOS ONE's style requirements, including those for file naming.

Response: Numerous formatting changes were made throughout the article in order to comply with the PLOS ONE style requirements. No content changes were made to satisfy these requirements, so we did not track these numerous formatting revisions.

2. We note that you have stated that you will provide repository information for your data at acceptance. Should your manuscript be accepted for publication, we will hold it until you provide the relevant accession numbers or DOIs necessary to access your data. If you wish to make changes to your Data Availability statement, please describe these changes in your cover letter and we will update your Data Availability statement to reflect the information you provide.

Response: The relevant DOI is available in the paper, in the first paragraph of Results.

——————— Response to Reviewer 1 ———————

Reviewer 1: The paper is well-written and the questions on identifiability posed at the beginning are important.

Response: Thank you.

Reviewer 1: However, all systems considered in examples have constant coefficients, hence presumably analytical solutions for the main dependent variable are available explicitly in terms of the unknown constant parameters. In reality, systems have non-homogeneous and/or nonlinearities. Can the approach proposed deal with such practical generalities of inhomogeneous and nonlinear systems?

Response: In this work, we demonstrate a method to find identifiable functions of parameters for a class of models commonly used in systems biology. In this class, the unknown parameters are assumed to be constant. There is an extensive amount of literature on the identifiability problem for this class of models, from linear compartmental models in pharmacokinetics, physiology, cell biology, ecology, and toxicology, to (bio-)chemical reaction networks assuming mass action kinetics such as phosphorylation systems or SIR models from epidemiology. While the reviewer is correct that there are many models where the parameters are considered non-constant, we limited our analysis to the constant parameter case. Our proposed approach cannot deal with non-constant parameters, as changing this assumption completely changes the approach for testing structural identifiability of such systems. In fact, testing the structural identifiability of systems with non-constant parameters remains an open problem.

Reviewer 1: There is some exaggeration of formalism in the first parts of the paper, which is actually not needed.

Response: We have stripped out some of the formalism that was unnecessary, e.g., a lengthy discussion of trace tests. If you see any other overly formal passages that could use our attention, please identify them.

Reviewer 1: Can the analysis be extended to PDE's?

Response: Our analysis is based on the differential algebra approach to structural identifiability, which is limited in scope to ODEs. One, in theory, could extend the differential algebra approach to PDEs, but that is beyond the scope of this paper.

Reviewer 1: The references should be written in the style of the journal and be consistent throughout.

Response: Done.

Reviewer 1: Several references are on arXiv, i.e. not reviewed yet, and limit the credibility of the citations.

Response: All of the references are now published or accepted for publication, and the paper has been updated to reflect this.

Reviewer 1: The word 'geometry' used is not fully justified and it could be misleading. Suggest removing and find a better formulation.

Response: The word "geometry" does not appear by itself in the manuscript. However, it is used in the context of the term "algebraic geometry" which is the branch of mathematics focused on solving polynomial systems. The subfield that focuses on numerical computations in algebraic geometry is called "numerical algebraic geometry." Since our methods utilize numerical computations to solve polynomial systems, "algebraic geometry" and "numerical algebraic geometry" are the proper mathematical terms.

Reviewer 1: Given a set of parameters that are desired to estimate and a governing set of equations, can you design the input-output data for identifiability?

Response: The question of finding the minimum number of input/output functions for a given model to become identifiable is still an open question. The question has been partially answered for the case of adding outputs only, as in Anguelova et al (2012), but the case of adding both inputs and outputs remains an open question. We do not address this problem, as we were concerned with testing identifiability and finding identifiable functions of parameters, and not the experimental design problem of finding such minimal input/output sets.

Reviewer 1: One can also consult the early works of Kitamura and Nakagiri on the identifiability of distributed systems.

Response: This reference is an interesting one on the identifiability of PDE systems. We checked this reference and it appears the authors are solving a different identifiability problem there, as their setup is very different from our setup. In particular, it examines the cases of distributed and pointwise measurements. The distributed measurement case seems to be a much stronger assumption on the form of the data than in our setup. The case of pointwise measurements is a similar problem, but their conclusions that identifiability conditions depend on the position of a detector and the form of the initial function or the input functions to the systems demonstrate that they are addressing a different problem. We do not believe that this reference is relevant to the particular problem we are addressing in this work.

——————— Response to Reviewer 2 ———————

Reviewer 2: I strongly recommend publication after the revisions suggested in the attached PDF. The techniques you provide establish identifiability results for models that would be too large for established methods. As such, it is a substantial contribution to the field of parameter identifiability analysis. Please see attached document for more details.

The article "Identifiability and numerical algebraic geometry" is an engrossing and insightful article applying state-of-the-art methods in numerical algebraic geometry to significantly enlarge our toolbox for parameter identifiability analysis. There are a few passages that should be clarified and some typos, but after minor revision, I strongly recommend publication.

Response: Thank you for you kind words, your suggestions, and your conclusion.

Reviewer 2: Edits:

- Abstract: “such as a mathematical” delete “a”.
  Response: Done.
- Abstract: Polynomial or rational ODE’s should be mentioned so that complex numbers make sense.
  Response: Added.
- P. 3: Monic is generally defined for univariate polynomials, so its use here should be clarified. Same for “non-monic”.
  Response: We have clarified that one can normalize so that one coefficient is 1. We also changed “non-monic” to “non-constant”.
- P. 4 (Method 1): The use of p as specializations of q is a bit unclear. At first it seems like q are themselves complex numbers.
  Response: This has been changed for clarity.
- P. 4: Explain how to interpret k_{ij} as flow from j to i.
  Response: The meaning of the parameter $\boldsymbol{k_{ij}}$ has been added.
- P. 4 (Figure 1): There are two in-edges to state 1 that do not have obvious interpretation.
  Response: Our caption to Figure 1 states that the input is represented by an arrowhead and the output is represented by a line segment with a circle at the end.
- P. 5 (Proposition 2.5): Is it necessary to specify the closure, rather than just the projection pre-closure? If so, you should specify Zariski (Euclidean, etc.) closure.
  Response: This has been updated, which points out equality when using closure in either Zariski or Euclidean topology.
- P. 7 (Method 2): Example 2.6 would have terminated at $r = 2$ based on the last line of this algorithm. Perhaps you need that the coranks are also constant from step to step.
  Response: Good catch – this has been updated.
- P. 8 (Definition 2.9): The use of “multi-valued functions” doesn’t quite work. In particular, the equation $\xi \circ c = g$ has a multi-valued fn on the LHS, and a single-valued on the RHS.
  Response: Good catch. We fixed the language in this definition and the subsequent example.
- P. 8 (Problem 2.11): The phrase “given by the functions...” is a bit unclear. Are these the elements of the tr.basis?
  Response: These functions were unnecessary and have been removed.
- P. 8 (Prop 2.12): Given this setup, wouldn’t f be *globally* identifiable? If not, can you give an illustrative example?
  Response: Thank you for this catch. We believe that you are correct, and a variation of this result is stated in Meshkat et al (2009), but we do not have a rigorous proof of this, so we are leaving it as “identifiable.”
- P. 9: The sentence beginning “While this information....” is difficult to parse.
  Response: Reworded.
- P. 11: For readers who are not familiar with the trace test, it will be difficult to understand from this description. In particular, the function $p_z(t)$ is not clearly defined, and it’s not clear that summation is coordinatewise. I would recommend including a clarifying picture (see, for example, Figure 1 in Leykin, Rodriguez, Sottile). Alternatively, skip an explanation of the trace test and just include the reference.
  Response: This is a good point. We have removed 18 lines and included the recommended reference.
- P. 13: Replace “yielding” with “yielding the result that” or “indicating that”.
  Response: Done.
- P. 13: “is the number of which correspond with the same input-output” $\rightarrow$ “is the number of points corresponding to the same input-output.”
  Response: Much better – done.
- P. 14 (Section 4, Paragraph 1): “every function of the parameter” $\rightarrow$ “parameters”
  Response: Oops – thanks.

- P. 15: In the sentence beginning “Hence”, the conclusion is not immediately obvious. Is it because all parameter values are different than in p?
  Response: Yes, this has been updated for clarity.
- P. 17 (Method 5): “Use homotopy sampling to additional” $\rightarrow$ “to find additional”
  Response: Oops – thanks.
- P. 21: “in these 16 unidentifiable parameters” (maybe add “in these latter 16” to differentiate from the first set.
  Response: Good point – thanks.
